# Public Health Professionals’ Perceptions on Intertwin Relationship in Multiple‐Birth Family Nursing

**DOI:** 10.1111/phn.70038

**Published:** 2025-11-12

**Authors:** Kristiina Heinonen, Tuulikki Trias, Jaakko Kaprio, Katri Vehviläinen‐Julkunen

**Affiliations:** ^1^ Department of Nursing Science University of Eastern Finland Kuopio Finland; ^2^ Centro Lapsi, centro de psiquiatría, psicoterapia y psicología perinatal Barcelona Spain; ^3^ University of Helsinki Institute of Life Science, University of Helsinki Finland; ^4^ Department of Nursing Science Kuopio University Hospital, University of Eastern Finland Kuopio Finland

**Keywords:** competence, intertwin relationship, midwife, multiple‐birth family, nurse, nursing, parenthood, twins

## Abstract

**Objective:**

To describe how dominance, submissiveness, and speaking role appear in the intertwin relationship between twins under 7 years old.

**Design and Sample:**

A cross‐sectional study design was used. The respondents (*n* = 72) were nurses. Information about the intertwin relationship was explored through Likert‐scale statements and open‐ended questions. This article reports the open‐ended questions that were analyzed by content analysis.

**Results:**

Dominance, submissiveness, and the speaking role appear in twins’ behavior. The twins take different roles, such as the space taker, the decision maker, the follower on the sidelines, the helper, or the recipient of help. Twins can interact as either being brave and visible or withdraw into the background. The quality of intertwin relationships manifests in daily life situations, which brings opportunities to observe, guide, and support the children's individuality and balance the intertwin relationship.

**Conclusions:**

To continue to support the growth and development of multiples in maternity and child health clinics, nurses need to have knowledge of the relationship between twins and the leadership, submissiveness, and speaking role associated with it. Work and guidance in nursing must be evidence‐based.

## 1 Introduction

Multiple‐birth family is a family with or expecting more than one same age child such as twins. Twins and triplets account for 2%–3% of births worldwide. Globally, more twins are being born than ever before (Ferriman et al. 2018; Monden et al. 2021). Multiple‐birth families belong to the vulnerable families. In comparison to singletons, multiples have unique challenges during conception, gestation, and birth, different kinds of health risks and developmental environments, as well as their individuality, all of which combine to impact and differentially affect family dynamics (Bryan 2005; Åkerman and Fischbein 2005; ICOMBO 2023).

The interaction between the parent and child is already established during pregnancy in a multiple pregnancy with more than one fetus and is strengthened after birth. The relationship between twins is usually close, special, and unique (Allen et al. 2020; Trias 2006, Tirkkonen et al. 2008; Segal and Knafo‐Noam 2021). It begins in the womb when they feel each other's presence and movements, and it continues after birth. Twins are either monozygotic (MZ) or dizygotic (DZ), sharing experiences, parental affection, and attention, and they are often close throughout life (Kaprio 2020; Penninkilampi‐Kerola et al. 2005; Trias 2006; Tirkkonen et al. 2008).

Multiple‐birth parents expected and needed more support in nursing care. Previous research shows that parents experience more stress and tiredness in multiple‐birth families than single‐child parents (Wenze et al. 2020; Lutz et al. 2012). Parents need information and support to see the twins as individuals and to give them individual attention. As part of this process, nurses may advise parents to devote enough time to each twin separately, talking to and maintaining eye contact with each individual. Such daily togetherness complements the time spent with both twins together (Heinonen 2022). The intertwin relationship and the company of may also compensate for the lack of maternal time and attention (Wang et al. 2022). Mutual dependence influences twins’ social interactions, leisure activities, and later, their somatic wellbeing (Trias 2006). The intertwin relationship and how to address it in supporting child growth and development, as well as parenting, are areas of specific multiple‐birth family knowledge (Heinonen 2013, 2022; Heinonen et al. 2024).

The identification of twins with each other is usually mutual, reciprocal, and equally strong (Leonard 1961). The relationship between MZ or genetically identical twins appears to be more solid (Trias 2006; Segal and Knafo‐Noam 2021; Taylor et al. 2021; Segal et al. 2023) and more likely to lead to deeper identification than in DZ twins. Interdependence between twins is common, but it is not the dominant feature of the interaction. The experience of interdependence is influenced by monozygoticism and sex (Penninkilampi‐Kerola 2006; Trias 2006; Kutschke et al. 2018). The co‐twin can be so significant that one child may only feel complete in the presence of the other. Each twin needs to individuate from the co‐twin (Allen et al. 2020; Segal and Knafo‐Noam 2020). Ebeling et al. (2003) address dominance and submissiveness in physical, psychological, and verbal dominance.

In a comparison of attachment to the mother between older siblings and twins, twin girls were found to have stronger attachment to the mother and the relationship of the twins with each other compensated for the lack of access to the mother and attention from her. Girls reported greater inter‐twin dependency than boys (Penninkilampi‐Kerola 2006; Wang et al. 2022). Interdependence can also compensate for negative experiences of interaction with a parent (Segal and Knafo‐Noam 2021). The ability to rely on the twin pair during difficult moments in life can maintain a twin's well‐being (Trias 2006). Taylor et al. (2021) highlight empathy, comforting, and giving and receiving mutual support in the relationship between twins. The intertwin relationship can sometimes develop too close and affect a child's health to such extent that they develop emotional and somatic symptoms such as stomach pain, sadness, and depression (Trias 2006, Tirkkonen et al. 2008). In late adolescence and early adulthood, twins leave the family home and generally move apart from each other, to eventually established their own families and adult lives (Wang et al. 2022; Wang et al. 2024).

Twins need more help than singletons to develop an individual identity. The twin identity includes both an individual dimension and a shared twin identity (Trias 2006, Tirkkonen et al. 2008). Parental interactions influence the twin identity, but it is also influenced by the twins themselves and by friendships (Trias 2006). The relationship between the twins, therefore, influences the development of both twins, but there is limited research evidence on this (Bryan 2003; Bacon 2006). The twin relationship is described as a single identity, which interferes with individual development and independence; a positive emotional bond, which makes it difficult to be separate; a shared identity, where opposing self‐concepts complement each other; an idealized twin, which makes it difficult to experience life as a separate individual; a competing identity, which can bring feelings of guilt; and a normal sibling relationship, which can be both fixed and distant (Schave and Ciriello 1983). Twins share the same developmental stage, and there is interdependence between them, while children of different ages differ (Penninkilampi‐Kerola et al. 2005). Siblings in the family have perceived the relationship between identical twins as closer than between other siblings (Greenwood 2018).

Parents’ individual need for information tends to increase as family dynamics evolve, particularly in situations where additional children are expected. In such contexts, it is essential to take into account the well‐being and adjustment of existing siblings (Burlingham et al. 2024; Beyers‐Carlson et al. 2022). The support provided to families with multiples can positively influence the development of children's emotional, cognitive, and physical skills (Burlingham et al. 2024). Special attention should be paid to the siblings in the family after the birth of twins (Burlingham et al. 2024; Volling 2012) Parents of multiple‐birth families have also expressed concern about not only twins but also the other children in the family after the birth of the multiples, and about the reduced attention and time the parent can give to the other children (Heinonen 2013).

The health professionals have pointed out their lack of information on multiple‐birth families and a need for further training. The parents of multiples expect and need more support and specific knowledge about parenting twins from nurses than they receive. To be able to support the parents of multiples, the growth and development of twins, and promote health and wellbeing, knowledge about the development and relationship between twins is needed (Heinonen 2013, 2019, 2022). This research is a part of our broader study in the TWIN LIFE 2021–2026 project Nurses’ competence in multiple birth family nursing. In this qualitative part of the study, information and observations are reported from the responses to open‐ended questions on the dominance, submissiveness, and speaking role in the relationship under 7‐year‐old twins.

## 2 Methods

### 2.1 Study Design

The study was conducted in a cross‐sectional design by questionnaire in maternal and child health clinics in 2022. The respondents (*n* = 72) were nurses, public health professionals such as midwives, public health nurses, and family care workers, with experience with multiple‐birth family nursing. Information about the intertwin relationship was explored through Likert‐scale statements and open‐ended questions. In the open‐ended research, questions were asked about how dominance, submissiveness, and speaking role appear in the relationship under 7‐year‐old twins. This article reports the open‐ended questions that were analyzed by deductive‐inductive content analysis (Elo and Kyngäs 2008; Holloway 2016; Kyngäs et al. 2020).

### 2.2 Participants and Recruitment

According to the information provided by the persons in charge of maternal and child health clinics, a total of 144 nurses out of 156 were invited to attend the meeting events. The information session consisted of a presentation by the principal investigator, the content of which consisted of the presentation of and participation in the study, the processing of personal data, and the grounds for processing, data protection, the significance of the research, the voluntary nature of participation, and informed consent. Participants were offered the possibility to ask questions, and they received the researcher's contact information. After each information session, the participants received an email with a link to the survey from their person in charge. The background information of the participants is described in Table 1.

**TABLE 1:** The participants' (*n* = 72) background information.

Determinant	*n*
**Age (years)** median (Md) 40 years	
Under 30	7
30–39	26
40–49	21
50–59	7
60 or over	9
Did not respond	2
**Education**	
Post‐secondary or other vocational qualification	9
Polytechnic	58
University‐level degree	4
Other, which	1
**Job title**	
Public health nurse	67
Midwife	1
Public health nurse and midwife	2
Family care worker	2
**Working area**	
Maternity clinic	2
Child health clinic	16
Maternity and child health clinic	53
Other, where?	10
*(women's health examinations, contraceptive clinic, student health care, population responsibility, special area)*	
**Years of work in the current position**	
Under 5 years	23
5–10 years	14
11–15 years	13
Over 15 years	22
**Multiple‐birth family as a client**	
Families of twins	71
Families of triplets	15
**Meeting multiple‐birth families**	
At the clinic	23
Home visit	5
At maternity and child health clinics and home visits	49
Elsewhere, e.g., at the reception	5
**Education/continuing education**	
Yes	4
No	68

### 2.3 Data and Analysis

**FIGURE 1:**
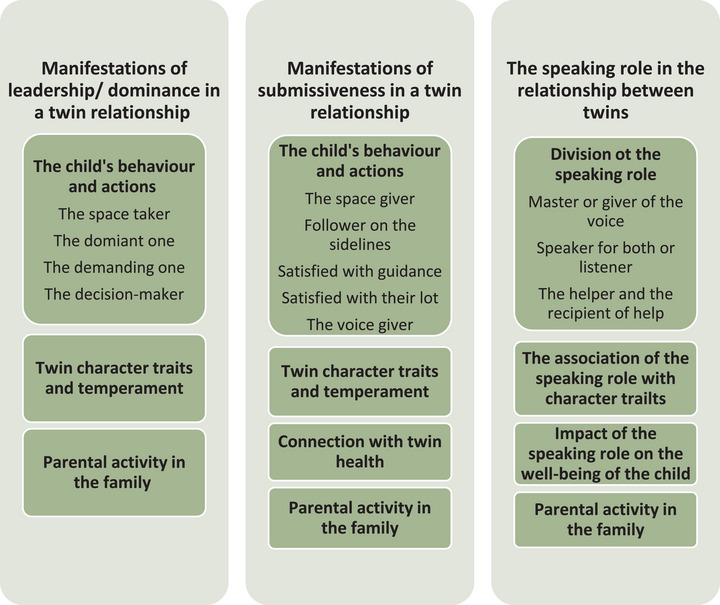
The manifestations of the intertwin relationship. [Colour figure can be viewed at wileyonlinelibrary.com]

The coded responses to the open‐ended questions were transferred from the web‐based RedCap to a Word file and analyzed on a question‐by‐question basis using deductive‐inductive content analysis by principal investigator (Elo and Kyngäs 2008; Holloway 2016; Kyngäs et al. 2020). A total of 72 respondents answered the questionnaire. About half of the respondents answered the open‐ended question about the intertwin relationship: leadership (*n*  =  34, 47%), submissiveness (*n*  =  37, 51%), and the speaking role (*n* =  34, 47%). Among the responses, 10 respondents did not know or had not heard of the issue, leaving 42% (*n*  = 30) for leadership, 46% (*n*  =  33) for submissiveness, and 46% (*n*  =  33) for the speaking role. The data consisted of responses written by participants in person. Deductivism in this study meant directing the research questions based on the findings of the previous study toward dominance, submissiveness, and the speaking role in the relationship between twins. Inductivity allowed for the incorporation of new information generated by the respondents into the research findings. We were interested in how dominance, submissiveness, and the speaking role appear in the intertwin relationship. The unit of analysis was the sentence and/or sentence fragment. The data were reduced and grouped in an overlapping way. Similar reduced expressions formed subcategories, which progressed to super‐categories, main categories, and a unifying category (Holloway 2016) Table 2. The progression of the analysis is described in Table 3. The manifestations of the intertwin relationship are presented in Figure 1.

**TABLE 2:** The result of the analysis subcategories, super‐categories, main categories, and a unifying category.

Unifying category	Complex factors and manifestations of the twin relationship		
Main categories	Manifestations of leadership in a twin relationship	Manifestations of submissiveness in a twin relationship	The speaking role in the relationship between twins
Super‐categories *Subcategories*	The child's behavior and actions *The space taker* *The dominant one* *The demanding one* *The decision‐maker* Character traits and temperament of the child *(connect to the temperament)* Parental activity in the family *(parents' activity)*	The child's behavior and actions *The space giver Follower on the sidelines* *Satisfied with guidance* *Satisfied with their lot* *The voice giver* Twin character traits and temperament *(connect to the temperament)* Connection with twin health *(twin's wellbeing)* Parental activity in the family *(parents' activity)*	Division of the speaking role *Master or giver of the voice* *Speaker for both or a listener* *The helper and the recipient of help* The association of the speaking role with character traits (speaking role) Connection with twin health *(twin's wellbeing)* Parental activity in the family *(parents' activity)*

**TABLE 3:** An example of analysis based on how dominance appears in an intertwin relationship.

Original	Reduction	Subcategory	Super‐category	Main category	Unifying category
“ … in play situations or other everyday situations where one twin takes space or adult attention from the other. For example, one is usually the initiator of play and the other's role is always to be a mere participant in the play.” “The leader doesn't give the other one the floor but speaks for both.”	‐ One twin takes space or adult attention from the other ‐ Does not give the other one the floor but speaks for both	The space taker	The child's behavior and actions	Manifestations of leadership in a twin relationship	Complex factors and manifestations of the twin relationship
“Often one thinks” and the other “does.” Leadership can appear when the one with a good idea makes the other ‘do’ things. “The leading child decides what to do. Can demand more attention.”	‐ One "thinks" and the other "does" ‐ Makes the other 'do' things ‐ Demand more attention	The dominant one			

### 2.4 Ethical Considerations

All participants were informed about the research and they obtained and signed an informed consent informed consent based on understanding of the research as stated in The Helsinki declaration (World Medical Association 2013). The participants were health professionals (nurses), so no prior ethical review was required (TENK 2023). Prior to data collection, a member of the Research Ethics Advisory Board was consulted regarding research in an indigenous area. The study complied with the provisions of the Data Protection Act (DPA) (1050/2018) and the EU General Data Protection Regulation (GDPR) (679/2016) for the processing of personal data. The personal data processed were minimised. The results of the research can be used to develop the skills of professionals and support parenting. The knowledge will be used to support twin growth and development, considering the specificities of the intertwin relationship. Ethical justification is provided by the limited evidence‐based knowledge, the need to develop nursing care and to support families with multiples (TENK 2023). An information was given session organised for the by the persons in charge of the organisations on the study, personal data collection, data protection, data storage and study life cycle, with an opportunity for questions before the voluntary decision to participate. The questionnaire begins with an information sheet on the study and an informed consent form. Where consent was missing (*n* = 4), responses were not used. The participants who asked for further information could not be identified or linked by the questionnaire.

## 3 Results

### 3.1 Manifestations of Leadership/Dominance in a Twin Relationship

#### 3.1.1 The Child's Behaviour and Actions

##### 3.1.1.1 The Space Taker

The taking of space became evident as one twin took the space and adult attention from the other twin. The space‐taker also came up with ideas for joint play and defined the roles of the twins. In the interaction between twins, space‐takers expressed themselves so that the twin in the speaking role took care of talking for both, and the other twin did not have time to express themself or have a turn to speak.

“ … in play situations or other everyday situations where one twin takes space or adult attention from the other. For example, one is usually the initiator of play and the other's role is always to be a mere participant in the play.” (15)

“The leader doesn't give the other one the floor but speaks for both.” (39)

##### 3.1.1.2 The Dominant One

Leadership involved being bossy, demanding, and leading. The assertiveness of leadership was reflected in the twins’ courage and sociability. Assertiveness was associated with different situations, such as implementing one's own ideas, choosing common play, and responding to different situations.

“Often one ‛thinks’ and the other ‛does’. Leadership can appear when the one with a good idea makes the other ‘do’ things.” (47)

“The older of the children will, for example, tell you in a vaccination situation whether situation makes them nervous or not. The more submissive twin may not be heard.” (27)

##### 3.1.1.3 The Demanding One

Being demanding was connected with perseverance in getting one's own way or getting an idea through. The sibling was demanded to do something or implement an idea. Demandingness was expressed in choosing toys and play, where the other twin was not heard in the same way.

“One of the children is stronger‐willed and better able to get their point across.” (10)

“The more outspoken individual insists on persevering, leaving the more submissive one to ‘wait their turn’, the bigger one, for example, takes control of the toys.” (37)

##### 3.1.1.4 The Decision‐Maker

Decision‐making was evident in one twin deciding on the activities of both twins, or at least making the final decision for both. Leadership in decision making was expressed in different areas, such as deciding on shared play, choosing toys and games, and forming opinions.

“One of the children always decides what to do and always ends up making the decisions in different situations.” (8)

“So the bigger of the two decides what the twins do and where they go, who the twins play with and who they don't play with. Might say out loud if they think the food they are offered is good or not and the other one is silent.” (23)

#### 3.1.2 Twin Character Traits and Temperament

The manifestation of leadership in the character traits of the child included a subclass of the connection with character traits. The leading twin was described as strong‐willed, controlling, and dominant. Leadership was associated with sociability and activity. It was more related to the child's temperament and personality traits than to the relationship between the twins.

“If the personalities are different, for example, shy and brave. The brave one speaks for the shy one and can lead games.” (56)

“Leadership is more about differences in temperament than specifically about the relationship between the twins.” (16)

#### 3.1.3 Parental Activity in the Family

The actions of the parents were seen to be related to the strengthening or weakening of the role of the twins in their relationship, but also to the impact on the well‐being of the twins. Leadership was perceived to strengthen the self‐esteem of the leading twin and to bring more courage.

“One is an active researcher and a go‐getter by nature and the other is a quiet thinker. One speaks and the other is quiet. The older one considers the other to be more courageous and brings this out in his words and actions, and so the other personality takes on the role of a follower.” (55)

“As an expression of will/desire and acceptance of the will/desire by the parent.” (24)

### 3.2 Manifestations of Submissiveness in a Twin Relationship

#### 3.2.1 The Child's Behaviour and Actions

##### 3.2.1.1 The Space Giver

Giving space was described as the submissive twin's voluntary withdrawal into the background and giving space to the other twin in different situations. The assumed role manifested itself in a natural way in the actions of the submissive twin. The submissive twin voluntarily waits for his turn and changes his behaviour so that the more demanding twin is given priority.

“The other waits on his own initiative so that the leader to has space.” (51)

“The more submissive party starts to act in such a way that the more demanding party is prioritized in the action.” (24)

##### 3.2.1.2 Follower on the Sidelines

The follower on the sidelines is present in situations but remains in the role of a follower and sympathiser. The follower on the sidelines allows the other twin to make more of the decisions, even if they also have a personal opinion. However, they seem to be content with the way matters are expressed by the other twin.

“… in a role where you let the other person to say more and go along with them a little bit in what they do and even just what their opinion is on something.” (12)

“The other twin can go along with the leader and remain on the sidelines in the role of a spectator and bystander.” (11)

##### 3.2.1.3 Satisfied With Guidance

The one who is satisfied with guidance is content to wait for instructions and their turn. Gradually, this twin becomes increasingly accustomed to doing what the other twin demands and waits for permission and instructions. The one who is satisfied with guidance waits and obeys, even if disagreeing, and gradually becomes willing to do what the other says.

“One decides and the other gets used to the other deciding.” (39)

“One twin obeys what the other says, even if they disagree.” (17)

“One of the twins is used to taking on their role by waiting for a turn and waiting for permission and instructions from the other twin…” (15)

“One is always ready to do what the other says.” (69)

##### 3.2.1.4 Satisfied With Their Lot

The one who is satisfied with their lot has adopted a certain model of acting in various recurring situations. This twin has given up and submits to the example of the leading twin, and instead of putting themself forward, keeps quiet. Submission in a situation of choice means coming last and settling for what is left. The one who is satisfied with their part does not resist or express a dissenting opinion or themself. This twin can also stop trying and remain on the sidelines in the role of follower. The withdrawn behaviour of the one who is satisfied with their part becomes apparent when both twins are present. One respondent highlighted the relationship between the twins, where one twin may assume a different role in the presence or absence of the other twin.

“Adapting to another role can be easier with the other twin out of the picture.” (15)

“Does as the other instructs, settles for what's left, chooses last.” (47)

“Is resigned to waiting for their turn, settles for what they get. Does not make a ”fuss“. One child submits to the will of the other.” (37)

##### 3.2.1.5 The Voice Giver

Giving up the voice meant that one twin was overshadowed by the other twin, also in terms of speaking and being heard. The voice giver is unable to make their voice heard and will hear and may not have the courage to express their own opinion. The voice giver follows suit, follows the example, and takes a cue from the other twin. Voice giving can also take place by mutual consent.

“One of the twins can be left in the role of letting the other one decides more and go along a little bit with them in what they do and even just in what opinion they form about something.” (12)

“The submissive twin is content to follow the example of the leading twin and not make their own opinion or feelings known. Doesn't argue back but follows. Doesn't dare to speak their own mind.” (67)

“One of the twins answers questions on behalf of the other.” (8)

#### 3.2.2 Twin Character Traits and Temperament

The manifestation of submissiveness in the child was associated with individual character traits. A submissive child was perceived to be more sensitive, shy, timid, and quieter than the sibling. The submissive child was seen as more reserved in different situations and more likely to take a back seat. This was evident in the relationship between the twins as well as in the relationship with outsiders.

“The more introverted of the twins may be more easily submissive to their sibling, the outside world and other people.” (50)

“One is shyer, the other is bolder… One may be slow to warm up to new things, the other more open…” (16)

#### 3.2.3 Connection With Twin Health

The effects of submissiveness on the child's well‐being were described as the child withdrawing and being left behind. A more submissive child was more likely to be quieter than their sibling. Submissiveness was associated with melancholy, possible challenges to the child's development, and even depression. Submissiveness could also be seen as the child giving up and not trying.

“The other does as the sibling instructs, takes a back seat, becomes quieter, even melancholy.” (58)

“The other child gives up and doesn't even try. The other may become depressed and withdraw. There can be different developmental challenges.” (29)

#### 3.2.4 Parental Activity in the Family

The participants described how parents' actions can either strengthen or weaken the role of the child in the relationship between the twins.

“One is an active researcher and go‐getter and the other is a quiet thinker. One speaks and the other is quiet. The older one considers the other to be more courageous and expresses this in his words and actions… in this way the other personality assumes the role of follower.”(55)

### 3.3 The Speaking Role in the Relationship Between Twins

#### 3.3.1 Division of the Speaking Role

##### 3.3.1.1 Master or Giver of the Voice

In the relationship between the twins, one twin was the master of the voice and the other the giver of it. The one with the dominant voice was better able to articulate and express themself than the sibling and was more oriented toward the role of the speaker. The one with the dominant voice was more sensitive to speaking for both of them and had a louder voice. The role of the dominant voice was more readily associated with the leading twin. Voice dominance meant that only one twin's voice was heard, and the other twin could not express themself.

“One of them is better at saying things and is more likely to be the speaker.” (69)

“ One speaks up and explains things for the sibling, decides the games, is louder. One child's voice is heard when the other is quiet.” (58)

##### 3.3.1.2 Speaker for Both or Listener

Speaking for both meant that one of the twins would speak and answer on behalf of both of them. The other twin did not have time to respond. This was also associated with the more advanced speech development of the one speaking on behalf of both and the slower speech development of the child who was submissive. The speaker for both clarifies and does not give the floor to the other and decides things on behalf of both. Speaking for both was described more in terms of leadership than submissiveness.

“One twin may start to speak for the other, in which case the other's voice literally may not be heard in the same way.” (11)

“One of the twins may be more social and talk about things and the other more quiet, remaining a listener.” (46)

##### 3.3.1.3 The Helper and the Recipient of Help

The helper in speaking handled the negotiations and speaking situations in a variety of ways. The twin in the speaking role helped the sibling to adapt to new situations, such as interacting with new people in situations outside the family. The helper managed negotiations and spoke for the other, taking care of both their own and the sibling's affairs. The helper also acted as a protector and interpreter for the sibling when necessary to reach and secure an understanding. Compared to the sibling, the helper role was associated with more advanced speech development and sociability.

“One speaks for the other, answers for the other, especially in situations outside the home, such as at day care.” (43)

“The speech of one is less developed, the other interprets the messages.” (55)

“The spokesperson is braver and protects the shyer sibling.” (53)

#### 3.3.2 The Association of the Speaking Role With Character Traits

The one taking on the speaking role was also bolder, more assertive, and more outgoing in different social situations than the sibling. The more sensitive and quieter child was also perceived to be overshadowed by the temperament of the sibling in the area of speaking. The twin who was more submissive in the speaking role was more withdrawn, responded more briefly, and was quick to hand over the floor to the sibling.

“The more outgoing sibling can be a pioneer and an advocate.” (50)

“The spokesperson says things for the other person, doesn't ask what the other person thinks, but decides for the other person.” (67)

“The more talkative one answers when the quieter one is asked or interprets the speech. ” (55)

#### 3.3.3 Impact of the Speaking Role on the Well‐Being of the Child

Being left in the background in an interaction situation meant, in particular, that the twin who was submissive in speaking was left aside. It also meant not being heard, self‐esteem issues, silence, and associated melancholy. The twin in the speaking role was found to have advanced speech development and social skills. There may also have been issues requiring a health assessment for the other twin in relation to delayed speech development.

“One is overshadowed by the other's opinions and actions, unable to make their voice and will heard.” (8)

“Self‐esteem problems maybe later.” (27)

“If one child is a better speaker than the other and more confident than the other or one child has a speech delay.” (10)

“Does as the sibling instructs, takes a back seat, becomes quieter, more melancholy.” (58)

## 4 Discussion

### 4.1 Review of the Results

This study formed part of wider research on multiple‐birth families and aimed to describe and explore how midwives, public health nurses, and family care workers working in a maternity and child health clinic experienced leadership, submissiveness, and the speaking role in the relationship between twins under school age. The study provides new insights into the manifestation of the intertwin relationship, particularly in the behaviour of twins and their actions in different daily situations, linked to perceptions of the child's personality traits and temperament. This study also contributes novel insights into the observed dynamics of twin relationships and broader family interactions. The information brought by the professionals is new and important. This knowledge can be used in supporting parenting and twin growth and development, and in the development of education and training.

The respondents described leadership in the intertwin relationship as reflected in the child's behaviour in different roles, such as taking space, dominating, demanding, and deciding. The child's character traits were strong‐willed and sociable. The dominant twin's character and temperament were classified as being outward‐looking, strong‐willed, determined, and active. We noted any mentions of a connection between dominance and twin health and well‐being. This is not only a psychological question, but also a developmental one. The actions of the parents can potentially strengthen or weaken dominance. It is important that parents are aware of dominance in the intertwin relationship because they have an important role in influencing the relationship. The parental influence on twins is significant, a point that will be further explored in later studies (Heinonen 2013; Trias 2006; Segal and Knafo‐Noam 2020, Segal and Knafo‐Noam 2021). Positive parental feelings and the creation of a positive atmosphere have been found to be associated with a positive twin relationship (Mark et al. 2017). Positivity has also been found to influence the experience of closeness and dependence between twins, and negativity to influence dependence, conflict, and competition between twins (Segal and Knafo‐Noam 2021).

Submissiveness in the intertwin relationship was expressed in terms of giving space, watching from the sidelines, being controlled, being content with their part, and giving up their voice. Some of the subclasses are very close, but differences can be found; twins who are content with being controlled gradually become more and more accustomed to accepting this and are content to do as the other twin wishes. However, they have their own opinion on matters. On the other hand, those who are dissatisfied with their part have already adopted a model of action in which the surrender of their own will and submission to the will of the other is more pronounced than in the case of those satisfied with being controlled when both twins are present. However, one respondent pointed out how the role of multiples can still be apparent in the absence of the other twin.

Some submissive twins appeared to be satisfied with their part and had clearly accepted their role. They would wait for a turn, as well as for permission and instructions from the other twin. Submissiveness is associated with the twin character and temperament in terms of being inward‐looking, sensitive, quiet, calm, and flexible, in other words, bending to the will of the other twin. Connections between the submissive role and twin health were mentioned, such as uncertainty, immaturity, and depression. The parents’ actions can potentially strengthen this role, as they may give more attention to the dominant twin and, at the same time, strengthen the other child's role of follower. The participants did not suggest that leadership, submissiveness, and the speaking role were related to the child's gender or twin type. Previous studies have highlighted this aspect, that is, leadership, submissiveness, and the speaking role also being related to the child's gender, such as physical leadership in boys (Ebeling et al. 2003) and psychological leadership and the speaking role in girls (Trias 2006; Tirkkonen et al. 2008).

In this study, child health and well‐being issues were rare in the case of the twin who assumed the role of leader. Several effects related to the subordinate twin were described, including withdrawal, aloofness, self‐esteem problems, melancholy, and depression. Respondents also described the follower role of the submissive twin, being compliant, giving up their voice, and even giving up trying. It may be that the impact of leadership on the health and well‐being of the leading twin is not yet recognised, or data are lacking. Trias (2006) observed that very strong leadership or submissiveness is reflected in the emotional life of twins. Tension and nervousness were somewhat more typical traits for leaders, while lower self‐esteem and physical symptoms were more common for submissive (Trias 2006). Previous studies have also demonstrated that the leading twin has more neurotic‐type symptoms during adolescence, school years, and young adulthood, which are also more obviously linked to the acceptance of responsibility for both them and their twin compared to the other twin (Ebeling et al. 2003; Trias 2006; Tirkkonen et al. 2008). The submissive twin has been found to have lower self‐esteem and psychosomatic symptoms such as abdominal pain and headaches. Submissiveness in psychological leadership increased depression, nervousness, and psychosomatic symptoms in boy and girl–boy twin pairs (Ebeling et al. 2003; Trias 2006). In child health care, knowledge about the twin relationship, its manifestation, and its impact on the child is needed to support the growth and development of children growing up as twins. This implies difficulty in being apart, in forming friendships, but also in basing one's interests on other personal choices. The nature, permanence, or change in the relationship between twins was not addressed in this study. Participants' observations suggest that the leading twin can influence the opinions and choices of both twins by being dominant, leaving the submissive twin without a voice, opinion, or will. Too close a relationship can make it difficult to form other friendships.

According to Ebeling et al. (2003), before and during school age, girls were more psychological and linguistic leaders than boys. Linguistically submissive boy twins had higher rates of depression and psychosomatic symptoms. They rarely have an opportunity to demonstrate their own abilities, skills, and opinions when following and accompanying their twin pair (Moilanen 1996). The dependency of the twins was found to be clearly related to their interactions with each other, interpersonal relationships, and leisure activities in adolescence and early adulthood. Furthermore, the individual experience of dependence was associated with psychological and psychosomatic symptoms in twins, especially in adolescence, when the twins' attempts at independence are at their strongest or when only one of them experiences dependence. Interdependence can be an expression of love and affection and is enhanced by the similarity of twins and their constant togetherness. However, the relationship between the twins changed with age and the difference disappeared in adulthood, when most twins felt equal in leadership (Penninkilampi‐Kerola 2006). Trias (2006) reported that the leadership of boys continued into adulthood, but the leadership of girls in the speech role disappeared. After spending time together in childhood, new friendships formed later in life can change the experience of dependency (Segal and Knafo‐Noam 2021).

The health care professionals reported having observed/observe/ are aware that parents may influence the behavior of twins as reinforcing or undermining activities, which may also be unconscious. Multiples grow up with a sibling or siblings of the same age. Multiple siblings usually have a very warm and close relationship with each other. However, it is important that each child grows up to be an individual who can cope with situations and life without his or her same‐age sibling. This is why it is important in a multiple‐birth family to give each child individual attention daily. In multiple‐birth families, especially when multiples are the only children in the family, it is sometimes common for the children to spend a lot of time together. Although this close bond can be a strength, the text emphasizes the importance of individual development and social diversity. When children only interact with peers of the same age (like their twin or triplet siblings), they may miss out on learning from older or younger children. Having role models of different ages can support more well‐rounded development, but also encourages social skills, independence, and exposure to different personalities and experiences.

To support parenting, parents need holistic information and concrete examples of how the intertwin relationship manifests itself and how to promote child well‐being and health. Situations where one twin is overshadowed by the other should be urgently addressed. Twins are close to one another and share experiences in childhood, adolescence, and later life. The mutual relationship is meaningful throughout the twins’ lives (Segal and Knafo‐Noam 2020, Segal and Knafo‐Noam 2021).

In previous research, parents have raised the issues of day care, early childhood education, and schooling for twins from the perspective of whether the twins should be in the same or a different group or class (Heinonen 2013). When considering the issue from the perspective of twins, more research is needed on whether the separation of twins or keeping them in the same group can be recommended. The individual solutions of a multiple‐birth family should be supported, but the parents should also be given information on which to base their solutions. Multidisciplinary cooperation and training should be strengthened. The individual needs of multiple‐birth families require individual solutions (Heinonen 2013; Gordon 2015).

A multiple‐birth family is a special family, which means that parenting and child rearing require knowledge that is appropriate for such a family (Heinonen 2013, 2022; Segal and Knafo‐Noam 2021). Little research is available on the relationship and interaction between the twins. Excessive dependency, leadership, submissiveness, or taking the speaking role can be factors that jeopardize a child's overall development and health. In addition, evidence‐based information is needed for social and health professionals, it is also needed for teachers in day‐care centers and schools, and for the training of professionals (Heinonen 2017; Segal and Knafo‐Noam 2021). Nurses' competence and multiple‐birth family knowledge have a key role to play in supporting and informing these families. In the current research, participants were midwives, public health nurses, and family care workers and 72% answered that they need more education and training. The results indicated that the respondents often lacked knowledge about the intertwin relationship but were able to clearly describe the manifestations of dominance, submissiveness, and the speaking role in this relationship. The lack of knowledge and expertise on multiple‐birth families creates difficulties in understanding and supporting them (Heinonen 2013, 2017, 2022; Turville et al. 2021). Nurses need to have more special knowledge of the intertwin relationship to provide support, share knowledge, and give advice to parents.  Education is needed to enable the development of multiple‐birth family expertise in family nursing care. Further multidisciplinary studies are required on intertwin relationships and their impact on children's health and wellbeing, education, and impact. Care and family work for multi‐birth families and evidence‐based practice should be developed in a multidisciplinary way in collaboration with education, training, and social and health service providers.

### 4.2 Reliability

The reliability of the open‐ended questions was examined from the perspectives of credibility, transferability, confirmability, and authenticity (Kyngäs et al. 2020). Credibility was enhanced by a purposive sample, where all the respondents had experience of caring for multiple‐birth families. Reliability is enhanced by the open description of the research process, the analytical process is tabulated, and the results section is carefully described with selected direct quotations, which also increases confirmability (Elo et al. 2022; Kyngäs et al. 2020). The small total number of responses may also indicate a lack of information, which was expressed by 10 respondents. The responses were descriptive but varied in length. The results can be applied in discussions with different families (Kyngäs et al. 2020). In the multiple‐birth family research, the intertwin relationship has also been examined using summary variable groups based on Likert‐scale statements, with the results demonstrating a lack of knowledge and of information among the participants (Heinonen et al. 2024). Less than half of the respondents answered the open questions, but the answers were descriptive, and the research questions were addressed. Reliability could have been increased by conducting interviews after the survey. The COVID‐19 pandemic may have had affected the response rate.

## 5 Conclusions

A multiple‐birth family has special needs and specific information needs that need to be understood in health and social care.

To continue to support the growth and development of multiples in maternity and child health clinics, midwives/public health nurses must have knowledge of the relationship between twins and the leadership, submissiveness, and speaking role associated with it. The guidance of multiple‐birth families requires special information needed in multiple‐birth family situations.

Evidence‐based nursing must be strengthened and developed in a multidisciplinary way in collaboration with researchers, practitioners, and educators. On a global level, collaboration is especially needed to develop maternity health care and lower mortality rates.

More research is needed on family dynamics in multiple‐birth families and on the relationships between multiples, from various perspectives.

## Conflicts of Interest

The authors declare no conflicts of interest.

## Data Availability

The data that support the findings are not available because this is one part of the larger research.
